# Foreign body ingestion as a cause of a common carotid artery pseudoaneurysm^☆^

**DOI:** 10.1016/j.bjorl.2016.03.010

**Published:** 2016-04-25

**Authors:** Erica Batista Fontes, Maria da Graça Caminha Vidal, Juarez Rode, Roger Bagolin da Silva, Anderson Kahlbeck

**Affiliations:** a Universidade Federal de Santa Maria (UFSM), Departamento de Cirurgia, Cirurgia de Cabeça e Pescoço, Santa Maria, RS, Brazil; b Universidade Federal de Santa Maria (UFSM), Departamento de Cirurgia, Cirurgia Cardiovascular, Santa Maria, RS, Brazil

## Introduction

Foreign body ingestion is a common accident in clinical practice. In spite of the generally benign natural course, it can be associated with increased morbidity when presented with complications, such as esophageal perforation.^1^ This is particularly uncommon and is a life-threatening situation. A rare consequence of esophageal perforation by a foreign body can be the penetration of a great vessel. When it occurs, the repercussion can be drastic, such as severe hemorrhage at the time of penetration or later with the formation of a pseudoaneurysm (PA).^1^, ^2^ In this report, we present a rare case of esophageal perforation with the development of a traumatic common carotid artery PA after fish bone ingestion.

## Case report

**Figure 1:**
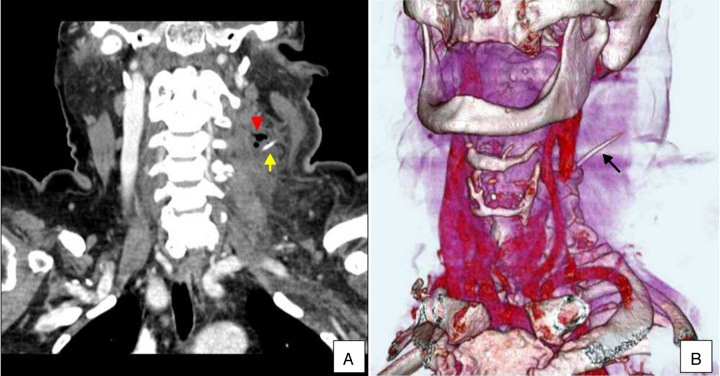
Contrasted CT scans revealing the presence of a foreign body (arrow) and gas consistent with an abscess (arrow head) in the left parapharyngeal region. (A) Coronal CT slice and (B) reconstructed 3D CT slices showing the fish bone lodged in the left parapharyngeal region (arrow).

A 71 year-old hypertensive and diabetic woman was referred to a tertiary hospital with a history of fish bone ingestion 7 days earlier. She presented with mild discomfort associated with a pricking sensation when swallowing, odynophagia and a mass on the left side of her neck. Examination of the neck revealed a painful, reddish, warm and edematous mass. Although there was no evidence of a pulsatile mass or bruit during auscultation, imaging was requested due to her history of fish bone ingestion. Plain and contrasted computed tomography (CT) scans revealed the presence of a foreign body in the left parapharyngeal/perihyoid region associated with an abscess, characterized by soft tissue infiltration and dissection through the cervical muscles (Fig. 1). Direct rigid endoscopy (DRE) did not reveal perforation of the esophagus or detect the foreign body.

**Figure 2:**
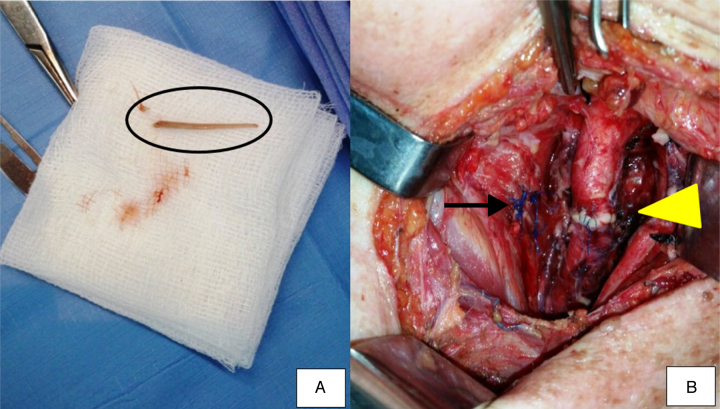
Surgical procedure. (A) In detail, the fish bone (black circle) which perforated the esophagus and caused a pseudoaneurysm to the left common carotid artery. (B) Sutured esophagus after removal of the fish bone (arrow) and end-to-end anastomosis of the left common carotid artery (arrow head).

Urgent exploratory surgery of the neck was performed by lateral cervical incision. To access the esophagus, the sternocleidomastoid muscle was temporally diverted. The fish bone was removed (Fig. 2A) and the area extensively washed with saline 0.9%. Perforation of the esophagus and a traumatic PA of the left common carotid artery were detected. After arterial clamping of the proximal and distal ends of the vascular lesion, the PA was excised and the common carotid artery was repaired with an end-to-end anastomosis (Fig. 2B) with no need of additional intervention on the internal and external carotid arteries.

The esophageal perforation was sutured and adjacent tissue was used to buttress the primary repair (Fig. 2B). The sternocleidomastoid muscle was dissected at its proximal insertions and used as a flap to protect the common carotid artery. Six Penrose drains were placed in the left paraesophageal area, and the surgical wound was sutured. A tracheostomy procedure and a Whipple gastrostomy was performed to nourish the patient and to prevent possible suture dehiscence. After surgery, the patient stayed 4 days in the intensive care unit and then was admitted to the hospital for another 15 days. During this period, a multidisciplinary approach was essential for her satisfactory post-operative progress. She was discharged home without any residual effects of the foreign body.

A contrasted esophagus CT and a CT angiography of the left common carotid artery were performed 37 days after the surgery at a follow up consult. The former exam revealed an unobstructed transesophageal lumen without extravasation of contrast agent. The latter showed a patent left common carotid artery with a normal caliber and the presence of calcifications on the carotid bulb. There was no evidence of fistulation and the patient presented satisfactory progress with no squeal.

## Discussion

In the presence of a foreign body in the carotid space, after ingestion and perforation of the esophagus, there is the potential risk of erosion of the common carotid artery during the migration of the object through the neck, which can cause massive hemorrhage, PA or aorto-oesophageal fistula. Therefore, its diagnosis and removal should be urgent.^1^, ^2^ Extracranial carotid arteries aneurysms and/or PAs can be asymptomatic or present as a pulsating cervical mass.^3^

Four retrospective studies were relevant when analyzing extracranial common carotid artery aneurysms and PAs. They revealed that the main causes of PAs were atherosclerosis, iatrogenic to post carotid procedures, trauma and others, such as, luetic and degenerative.^4^, ^5^, ^6^, ^7^ Three of these studies revealed that trauma was the least common cause when compared to arthrosclerosis and post carotid procedure.^4^, ^5^, ^6^ In the one study where trauma had a similar rate as to previous carotid endarterectomy, all traumatic PAs involved blunt injuries to the neck.^7^ Moreover, in none of these studies were fish bones the cause of carotid PA. Thus, the collected data corroborated to the singularity of the present report, with ingestion of a foreign body and not blunt trauma or iatrogenesis being the underlying mechanism for a common carotid artery PA.

When analyzing traumatic etiologies blunt and penetrating trauma to the neck corresponds to the major causes of PA.^7^ This condition can occur due to weakness of the vascular wall caused by the action of external components. The present study describes a rare case in which a foreign body perforated the esophagus, migrated through the adjacent tissue and penetrated the left common carotid artery resulting in a PA. Such circumstances were only previously reported in a few other studies.^1^, ^2^, ^3^, ^8^, ^9^, ^10^

Treatment options for common carotid artery PAs include clinical monitoring, surgical repair, endovascular interventions, or combined therapy. Surgical intervention is generally indicated when a common carotid artery aneurysm is detected mainly because of its potential for severe complications.^5^, ^6^, ^7^ There are five options for surgical treatment: aneurysm clipping, resection and end-to-end anastomosis, resection with an interposition graft, extracranial to intracranial bypass, and carotid artery ligation. Ligation of the vessel can be performed as a life-saving measure,^4^ although this technique is in disuse due to its high morbidity.^3^ The endovascular approach has been extensively studied and is currently accepted as an alternative in treating carotid artery aneurysms.^5^ However, although endovascular treatment has gradually gained recognition, the familiarity with the open surgical procedure, the emergency nature of the present case and the presence of infection excluded this strategy as a primary option. The endovascular approach could be an option as a temporary measure before surgery; but it would have left the esophageal perforation untreated and therefore increased the risk of mediastinitis and stent graft infection. Therefore, despite the increasing role of endovascular therapy in treating vascular disease, open surgical repair remains the most appropriate approach for extracranial carotid artery aneurysms.^2^, ^7^

The multidisciplinary approach to the treatment of a common carotid artery PA due to foreign body ingestion is essential for satisfactory patient outcome. Although the vascular surgeon can adequately treat the vascular disease, other treatments, such as, confection of a muscle flap to protect the manipulated carotid artery and drainage of the abscess may be executed by a head and neck surgeon or an otolaryngologist.^2^ Furthermore, pre-surgical planning and post-surgical care also account for adequate results. CT and contrast-enhanced CT is important as it assists in the detection of the foreign body and provides information on the affected structures, which will be important for the surgeon.^2^ Vascular contrasted imaging allows to assess the patency of the common carotid artery and to detect the presence of a possible fistulous path as well. In the present study the patient was well cared by a nurse, nutrition, occupational therapy, psychology and social assistance team, which was fundamental for the positive result.

## Conclusion

A rare case of esophageal perforation due to fish bone ingestion followed by migration of the foreign body through the soft tissues of the neck and the formation of a left common carotid artery PA was described in this report. The denouement of such disorder can be drastic; therefore, it is crucial to maintain a high index of suspicion in order to provide rapid diagnosis and to adequately prevent or treat possible outcomes. Moreover, due to the relatively small number of these types of cases reported in the literature, it is difficult to produce clear guidelines for how this condition should be managed.

## Conflicts of interest

The authors declare no conflicts of interest.

## References

[1] Karim H.H., Saikia M.K., Mitra J.K., Medhi J. (2015). Impacted fish bone leading to rapidly growing common carotid artery pseudoaneurysm. Indian J Vasc Endovasc Surg.

[2] Wang S., Liu J., Chen Y., Yang X., Xie D., Li S. (2013). Diagnosis and treatment of nine cases with carotid artery rupture due to hypopharyngeal and cervical esophageal foreign body ingestion. Eur Arch Otorhinolaryngol.

[3] Jean Roger M.T., Marcus F., Emmanuel F., Boniface M., Georges J.A. (2015). Pseudoaneurysm of the common carotid artery in the infant due to swallowed fish bone. Case Rep Radiol.

[4] Sabrout R.E., Cooley D.A. (2000). Extracranial carotid artery aneurysms: Texas Heart Institute experience. J Vasc Surg.

[5] Zhou W., Lin P.H., Bush R.L., Peden E., Guerrero M.A., Terramani T. (2006). Carotid artery aneurysm: evolution of management over two decades. J Vasc Surg.

[6] Dehn T.C., Taylor G.W. (1984). Extracranial carotid artery aneurysms. Ann R Coll Surg Engl.

[7] Garg K., Rockman C.B., Lee V., Maldonado T.S., Jacobowitz G.R., Adelman M.A. (2012). Presentation and management of carotid artery aneurysms and pseudoaneurysms. J Vasc Surg.

[8] Tang I.P., Singh S., Shoba N., Rahmat O., Shivalingam S., Gopala K.G. (2009). Migrating foreign body into the common carotid artery and internal jugular vein – a rare case. Auris Nasus Larynx.

[9] Gaudin M., Couchet G., Carrieres C., Ribal J.P., Rosset E. (2014). Finding a needle in the common carotid artery 3 years after ingestion. Ann Vasc Surg.

[10] Butler M.F. (1958). Perforation of the common carotid artery by a foreign body in the pharynx. Lancet.

